# Optimizing Inference Distribution for Efficient Kidney Tumor Segmentation Using a UNet-PWP Deep-Learning Model with XAI on CT Scan Images

**DOI:** 10.3390/diagnostics13203244

**Published:** 2023-10-18

**Authors:** P. Kiran Rao, Subarna Chatterjee, M. Janardhan, K. Nagaraju, Surbhi Bhatia Khan, Ahlam Almusharraf, Abdullah I. Alharbe

**Affiliations:** 1 Artificial Intelligence, Department of Computer Science and Engineering, Ravindra College of Engineering for Women, Kurnool 518001, India; 2 Department of Computer Science and Engineering, Faculty of Engineering, MS Ramaiah University of Applied Sciences, Bengaluru 560058, India; subarna.cs.et@msruas.ac.in; 3 Artificial Intelligence, Department of Computer Science and Engineering, G. Pullaiah College of Engineering and Technology, Kurnool 518008, India; m.janardhan0105@gmail.com; 4 Department of Computer Science and Engineering, Indian Institute of Information Technology Design and Manufacturing Kurnool, Kurnool 518008, India; knagaraju@iiitk.ac.in; 5 Department of Data Science, School of Science, Engineering and Environment, University of Salford, Salford M5 4WT, UK; 6 Department of Electrical and Computer Engineering, Lebanese American University, Byblos 13-5053, Lebanon; 7 Department of Business Administration, College of Business and Administration, Princess Nourah bint Abdulrahman University, P.O. Box 84428, Riyadh 11671, Saudi Arabia; aialmusharraf@pnu.edu.sa; 8 Department of Computer Science, Faculty of Computing and Information Technology, King Abdulaziz University, Rabigh 21911, Saudi Arabia

**Keywords:** adaptive partitioning, explainable AI, kidney tumor segmentation, optimization, weight pruning, UNet-PWP, DeepLabV3+, GCAM-attention

## Abstract

Kidney tumors represent a significant medical challenge, characterized by their often-asymptomatic nature and the need for early detection to facilitate timely and effective intervention. Although neural networks have shown great promise in disease prediction, their computational demands have limited their practicality in clinical settings. This study introduces a novel methodology, the UNet-PWP architecture, tailored explicitly for kidney tumor segmentation, designed to optimize resource utilization and overcome computational complexity constraints. A key novelty in our approach is the application of adaptive partitioning, which deconstructs the intricate UNet architecture into smaller submodels. This partitioning strategy reduces computational requirements and enhances the model’s efficiency in processing kidney tumor images. Additionally, we augment the UNet’s depth by incorporating pre-trained weights, therefore significantly boosting its capacity to handle intricate and detailed segmentation tasks. Furthermore, we employ weight-pruning techniques to eliminate redundant zero-weighted parameters, further streamlining the UNet-PWP model without compromising its performance. To rigorously assess the effectiveness of our proposed UNet-PWP model, we conducted a comparative evaluation alongside the DeepLab V3+ model, both trained on the “KiTs 19, 21, and 23” kidney tumor dataset. Our results are optimistic, with the UNet-PWP model achieving an exceptional accuracy rate of 97.01% on both the training and test datasets, surpassing the DeepLab V3+ model in performance. Furthermore, to ensure our model’s results are easily understandable and explainable. We included a fusion of the attention and Grad-CAM XAI methods. This approach provides valuable insights into the decision-making process of our model and the regions of interest that affect its predictions. In the medical field, this interpretability aspect is crucial for healthcare professionals to trust and comprehend the model’s reasoning.

## 1. Introduction

**Figure 1:**
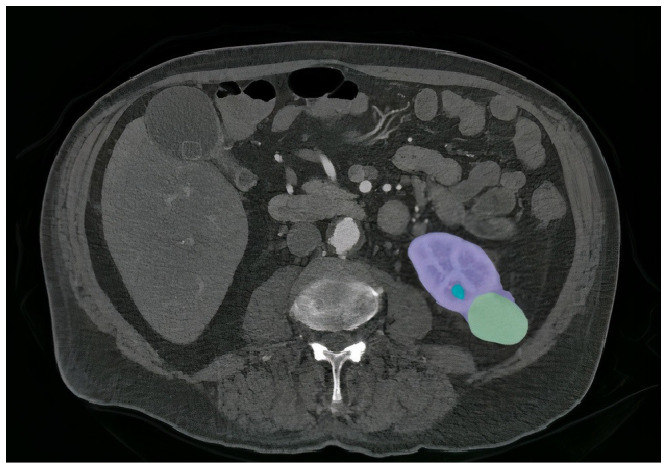
An example of a segmented slice from volume of CT scan Modality. The kidney region is shown in purple, the tumor is shown in green, and the cyst is shown in blue.

The kidneys serve a vital role in the human body by filtering waste products and toxins from the bloodstream [1,2]. Tumors, or cancers, result from the abnormal growth of cells and can manifest differently in individuals, leading to various symptoms. Early detection of kidney tumors (KT) is paramount for mitigating the risk of disease progression and preserving the patient’s life [2,3]. Although approximately one third of KT cases are identified after spreading to other areas, many remain asymptomatic and are incidentally discovered during unrelated medical evaluations. Kidney tumors can manifest as masses, cysts, or abdominal discomfort in patients, often unrelated to kidney function [4,5]. Nevertheless, some subtle symptoms or complications may arise due to KT, including low hemoglobin levels, weakness, vomiting, abdominal pain, hematuria (blood in urine), or elevated blood sugar levels. Anemia is also a common occurrence, affecting about 30% of KT patients [6,7]. Unfortunately, tumors and solid masses that develop within the kidneys frequently become cancerous. Detecting the presence of cancer is crucial in selecting the appropriate treatment method, as the prognosis and recovery rate often hinge on early identification. Computed tomography (CT) scans of the abdomen and pelvis are among the essential diagnostic tests used to ascertain the presence of kidney tumors. These scans provide specific characteristics that aid in tumor detection and assessment. Figure 1 illustrates a case of KT, depicting a renal mass lesion in the left kidney measuring approximately 4 cm (with the kidney in red and renal cancer in green). Given the life-threatening nature of tumors, accurate diagnosis is paramount, leading to various procedures aimed at assisting the physician [8,9]. Deep learning (DL) is a remarkably potent machine learning technology capable of autonomously acquiring numerous features and patterns without human intervention [10,11,12]. DL has empowered the development of predictive models for early tumor disease detection, with scientists relying on established pattern analysis techniques. DL algorithms have demonstrated superiority over traditional machine learning methods, yielding impressive results [13,14,15]. Furthermore, DL frequently achieves performance levels that match or exceed human capabilities, making it the preferred approach for handling image-related tasks [16,17]. This heightened recognition of DL in image processing, particularly within the medical domain, is attributed to the central role of radiology in extracting valuable insights from images.

Semantic segmentation, a task in computer vision, has witnessed significant advancements with the proliferation of DL techniques. DL has proven highly effective in enhancing image understanding [18,19]. These DL methods for semantic segmentation can be categorized into several approaches, including region-based, fully connected network FCN-based, and semi-supervised methods. Region-based methods follow a pipeline approach, initially extracting free-form regions from input images, followed by region-based classification. Ultimately, these methods assign labels to pixels based on the scored areas [19]. In contrast, FCN-based methods do not require region proposal extraction. Instead, they learn a direct mapping from pixel to pixel, allowing them to handle images of arbitrary sizes [19]. Semi-supervised methods are useful when dealing with datasets requiring extensive time for mask annotation. These methods aim to make the most of the available annotated data while incorporating unsupervised techniques to improve segmentation results [19].

Moreover, in addition to these primary categories, explainable artificial intelligence (XAI) [20] holds promise in shedding new light on disease characteristics, potentially serving as an indicator for assessing responses to exposure or other therapeutic interventions. Nevertheless, it is imperative that XAI offers clarity regarding the comprehensibility of its decisions, explanations, and potential associated errors. Therefore, before XAI can be considered a valuable and reliable tool for testing research hypotheses or aiding clinical decision-making, it must navigate several critical “translational gaps” [20,21]. Furthermore, the recently implemented European Medical Device [22] Regulation (EU MDR) imposes stringent transparency regulations that must be followed before integrating such a tool into clinical practice [23]. XAI thus holds the potential to be a pivotal factor in promoting greater transparency, ethical considerations, unbiased practices, and overall safety and trustworthiness in the deployment of DL algorithms within clinical settings.

Furthermore, in addition to our proposed model architecture, we have also incorporated state-of-the-art networks into our research; notably, the DeepLab V3+ [23] network along with XAI Grad-CAM [20]. Finally, our model’s performance has been rigorously assessed and validated using renal CT scans obtained from the KiTS datasets for the years 2019, 2021, and 2023 [24,25].

### 1.1. Contribution of Our Proposed Work

- Novel Methodology: We propose a novel methodology for medical image segmentation, addressing hardware constraints through adaptive partitioning and weight pruning.
- Progressive Model Construction: Our approach allows us to incrementally deepen UNet submodels while maintaining a consistent number of parameters, maximizing the architecture’s potential.
- GCAM-Attention:GCAM-Attention Fusion contributes to a model that excels in segmentation accuracy and computational efficiency and provides transparency and interpretability.
- Enhanced Kidney Tumor Segmentation: Our work focuses on kidney tumor segmentation, significantly improving accuracy and efficiency in this medical task.

### 1.2. Organization of the Paper

The remainder of this paper is organized as follows: Section 2 encompasses an exploration of related works, offering insights into the existing body of knowledge within the field. It provides a context for the current study by examining prior research endeavors. Section 3 delves into the methodology and materials employed, elucidating the architecture, dataset, and evaluation metrics that underpin our investigation. Section 4 lists the experimental outcomes and their meticulous analysis. The results are presented comprehensively, followed by an insightful exploration of their implications and significance. Concluding our discourse, Section 5 encapsulates the culmination of our study through the presentation of conclusions drawn from the research.

## 2. Related Works

Despite the numerous traditional CT image segmentation techniques proposed over the past few decades, including manual, threshold-based, atlas-based, graph-based, and hybrid methods, they exhibit limitations in accurately delineating kidneys in CT images. For instance, straightforward approaches like threshold segmentation are highly noise-sensitive and need help handling significant intensity variations in CT scans. Notably, both atlas-based and threshold-based methods require manual intervention and are susceptible to segmentation performance variations due to inter-rater differences.

Ronneberger et al. [26] employed the UNet model for medical image segmentation during the 2015 ISBI competition [26]. However, their approach utilized only a modest dataset of 30 images and data augmentation strategies, achieving a relatively modest error rate and clinching victory in the ISBI competition. Subsequently, various UNet-based algorithms, with adaptations and enhancements, gained prominence across diverse image processing domains consistently yielding commendable results.

In 2021, Heller et al. [27] summarized the top-performing methods in the KiTS19 challenge [23,24,25]. Notably, the segmentation models of the top contestants were all based on the UNet architecture. Fabian et al. [28] secured the first position with a 3D UNet-based approach, achieving impressive dice scores of 0.974 and 0.851 for kidney and tumor segmentation, resulting in a composite score of 0.912 [29]. Several other researchers [30,31,32,33,34] proposed kidney and tumor segmentation methods, achieving notable results in subsequent studies.

In recent times, researchers have increasingly turned to XAI to perform comprehensive assessments and provide explanations for model outcomes. For instance, Yang et al. [35] employed 3D Convolutional Neural Networks (CNNs) to classify Alzheimer’s disease while also offering visual explanations for their model’s decisions. Wickstrom et al. [36] utilized Gradient boosting (GB) techniques to improve the explainability of colon polyp classifications. Esmaeili et al. [37] integrated an explainability method based on Grad-CAM into the 2D glioma segmentation task. Saleem et al. [38] extended similar approaches to the realm of 3D image analysis.

Natekar et al. [39] harnessed Grad-CAM to shed light on the process of brain tumor segmentation, providing insights into the model’s decision-making process. Adebayo et al. [40] conducted a sanity check and discovered that class activation mapping (CAM)-based methods offer superior performance in classification tasks. Pereira et al. [41] put forward an explainability methodology that combines global and local information to enhance tumor segmentation, employing both GB and CAM techniques in brain tumor detection. Their experiments revealed that GB excels at identifying critical areas rather than categories, whereas CAM performs admirably in both tasks.

Furthermore, Narayanan et al. [42] utilized GoogLeNet and ResNet to detect various medical conditions such as malaria, diabetic retinopathy, brain tumors, and tuberculosis across different imaging modalities. They leveraged class activation mappings to provide visualizations that enhance the comprehension of these deep neural networks’ decisions. Moving forward to the KiTS21 challenge [25], Shen et al. [33] employed the COTRNet model for kidney segmentation, achieving a kidney dice score of 0.923. Adam et al. [25] used a 3D U-ResNet method for kidney segmentation and reached the 12th position in KiTS21. Zhao et al. [24] secured the first position in KiTS21 with a nnU-Net-based framework, attaining remarkable dice scores for kidney, mass, and tumor segmentation.

In conclusion, while various approaches have been explored for kidney segmentation, most kidney tumor segmentation studies rely on cascaded architectures as their primary models. However, 3D models demand significant computational resources, while 2D models may need more crucial spatial information. This paper introduces a novel segmentation approach for kidneys and tumors to address the computational complexity associated with 3D CNNs while maintaining high segmentation accuracy. The goal is to enhance the neural network architecture without compromising accuracy, presenting a versatile methodology applicable to kidney tumor segmentation. Beyond just KiTS19, KiTS21, and KiTs23 [24,25], our focus extends to aiding physicians in the rapid diagnosis of patients through improved segmentation results.

## 3. Materials and Methods

This section presents the methodology employed for kidney tumor segmentation using the KiTs variant dataset [25]. Our approach harnesses the power of deep neural networks, specifically UNet, combined with XAI, adaptive partitioning, and weight-pruning techniques to achieve accurate and validate the segmentation of kidney tumors.

### 3.1. Data Pre-Processing

**Figure 2:**
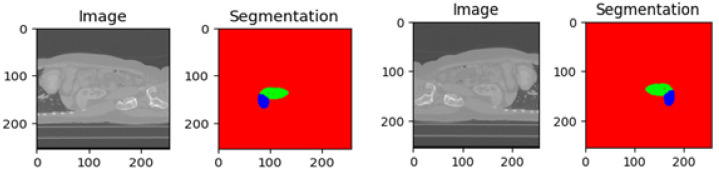
Visualization of kidney and tumor region segmentation using deep learning semantic segmentation.

The evaluation of kidney tumor segmentation techniques often leverages the KiTs 19, 21, and 23 variant datasets [25], a well-established benchmark for assessing the efficacy of such methodologies. This dataset comprises high-contrast CT images [2] acquired between 2010 and 2020 at the University of Minnesota Medical Center [2]. It encompasses data from 489 patients who underwent partial or radical nephrectomy for one or more kidney tumors. The dataset offers a rich diversity of scans featuring varying in-plane resolutions (ranging from 0.437 to 1.04 mm) and slice thicknesses (ranging from 0.5 to 5.0 mm). Each instance within this dataset is accompanied by ground-truth masks representing malignant tumors and healthy kidney tissue, as depicted in Figure 2. The meticulous creation of these masks involved the collaboration of medical students guided by expert radiologists—notably, the manual annotation process utilized solely the axial projections of the CT images. The dataset adheres to the NIFTI format and is defined by dimensions specifying the number of slices, height, and width. It has garnered widespread recognition as a standard benchmark for evaluating kidney tumor segmentation approaches, including the model proposed in this study.

In addition to the KiTS23 dataset [23,24], variants such as KiTS19 [23] and KiTS21 [24] play a crucial role in refining the evaluation process. Including these variant datasets enriches the evaluation process by capturing a broader spectrum of challenges and scenarios encountered in clinical practice. This comprehensive assessment not only strengthens the validation of the proposed model but also underscores its potential to address the complexities inherent in kidney tumor segmentation tasks.

### 3.2. Enhancing Kidney Tumor Segmentation: UNet Partitioning, Weight Pruning, and GCAM-Attention Fusion

The proposed model begins by constructing a complex Standard UNet model [28] for kidney tumor segmentation with deeper layers to extract the features. Let *X* represent the input CT scan [23,26] image, and *Y* be the corresponding ground-truth segmentation mask. The UNet model takes *X* as input and produces pixel-wise predictions $\hat{Y}$ for kidney tumor regions. The output of UNet is expressed as in Equation (1)

(1) $$\hat{Y}=UNet\left(X\right)$$

#### 3.2.1. Adaptive Partitioning for Scalable Submodels

The 3D-UNet architecture [26] is a highly intricate model with numerous layers, depth, and ten million parameters. Due to its complexity, it can be challenging to fit into standard GPU configurations. To overcome this limitation, an adaptive partitioning approach is employed to evaluate the intricacy of each UNet layer based on the interplay between the number of learnable parameters and computations performed during inference. This evaluation guides the division of the UNet into submodels, each with its own depth. Within this context, the complexity of each layer is described by the balance between the number of learnable parameters ($P_{i}$) and the computations carried out during inference via floating-point operations ($FLOPs_{i}$).

(2) $$\mathrm{Complexity}\;\mathrm{of}\;\mathrm{layer}\;L_{i}=P_{i}\times FLOPs_{i}$$

The parameter $max_{c}$ denotes the upper bound on the allowable complexity for each submodel, and $max_{p}$ signifies the envisaged number of partitions. Through mathematical analysis, we determine the target complexity $target_{c}$ as elucidated by the formula:

(3) $$target_{c}=\frac{\mathrm{Total}\;\mathrm{Complexity}}{max_{p}}$$

Target complexity ($target_{c}$) is computed to ensure each submodel balances complexity and resource constraints. This process results in smaller, more manageable portions of the original UNet, each designed to fit within standard GPU memory limitations.

#### 3.2.2. Weight Pruning for Efficient Resource Utilization

To enhance submodel efficiency, we employ a technique known as weight pruning, as referenced in [43,44,45]. This involves selectively reducing the number of parameters within a submodel by setting specific weight values to zero. By doing so, we can improve computational efficiency while also simplifying the submodel’s structure and preserving its ability to capture critical data features.

To provide further insight, let us consider a submodel represented by a set of parameters denoted as *W*. Weight pruning, as described in [46], identifies less impactful parameters based on their magnitudes. We can prune by assigning a value of zero to $W_{ij}$ for specific neurons, resulting in a sparser weight matrix. This process occurs after the incremental layer addition and fine-tuning phases. To balance model complexity and performance, we iteratively prune less influential parameters and fine-tune the submodel. This results in a more concise, resource-friendly submodel. Our methodology aims to optimize deep neural networks for practical use cases, particularly those with limited computational resources. This approach has significantly contributed to the effectiveness of our progressively trained UNet submodels in tasks such as biomedical image segmentation.

#### 3.2.3. Gradient-Weighted Class Activation Mapping(Grad-CAM)

Grad-CAM is an explainable AI technique designed for convolutional neural networks (CNNs) to visualize the regions in an input image that are important for the network’s classification decision [47,48,49]. Grad-CAM generates heatmaps that highlight the most relevant areas in the image, making it easier to understand the model’s focus and reasoning. Assuming a CNN model as *f* and an input image *x*, the goal of Grad-CAM [40] is to generate a heatmap that highlights the important regions in the image for the predicted class *c*. Grad-CAM follows these steps:

1. Identify the target layer: Grad-CAM focuses on the last convolutional layer of the CNN, which contains the high-level features that are most relevant to the classification task. Let *A* be the activation map of this layer with dimensions H×W, where *H* and *W* are the height and width of the map, respectively.
2. Compute the gradients: Calculate the gradients of the score for the predicted class *c* (denoted as $Y_{c}$) with respect to the activation map *A*. The gradients ($\frac{\partial Y_{c}}{\partial A}$) represent the importance of each activation for the predicted class.
3. Calculate the weights: Compute the weights α by global average pooling the gradients over the height and width dimensions [6].
  (4) $$\alpha_{k}=\frac{1}{H\;\cdot \;W}\sum_{i=1}^{H}\sum_{j=1}^{W}\frac{\partial Y_{c}}{\partial A_{i,j,k}}$$
  where *k* is the index of the *k*-th feature map, and $A_{i,j,k}$ is the activation at location (i,j) of the *k*-th feature map.
4. Compute the weighted activation map: Multiply each feature map in *A* by its corresponding weight $\alpha_{k}$, and sum the weighted feature maps to obtain the weighted activation map *L*.
  (5) $$L=\sum_{k}\alpha_{k}A_{k}$$
5. Generate the heatmap: Apply a ReLU function to the weighted activation map *L* to eliminate the negative values and obtain the final heatmap *H*.
  (6) H=ReLU(L)

The resulting heatmap *H* highlights the regions in the input image [49] that contributed the most to the predicted class *c*. Grad-CAM can provide insights into the model’s decision-making process, enabling users to identify potential biases, verify the model’s focus on relevant features, and ensure that the model does not rely on irrelevant or spurious patterns. Grad-CAM is specifically designed for CNNs and may not be applicable to other types of neural networks or machine learning models [23]. However, it has been widely used for explainability in image classification tasks and can be adapted for other tasks such as object detection or semantic segmentation.

### 3.3. Generating Attention Heatmap

The attention heatmap visualization technique highlights the regions within an input kidney tumor CT scan that receive the most focus from a neural network during the segmentation process. The generation of an attention heatmap begins with an input image represented as a 2D array with dimensions H(height) and W(width). In this process, a predefined center of attention, indicated by the coordinates $\left(attention_{c}enter_{x},attention_{c}enter_{y}\right)$, plays a pivotal role. Initially, an empty attention heatmap, denoted as *A*, is created with dimensions matching the input image’s dimensions. Subsequently, a Gaussian filter is applied to the attention heatmap *A*. This filter emphasizes the regions of interest surrounding the designated center of attention [50], and its extent is determined by a specified standard deviation (σ). In the next step, the Gaussian filter operation is applied as $A^{\prime }=G\sigma *A,$ where $A^{\prime }$ represents the filtered attention heatmap and Gσ is the Gaussian filter. Following applying the Gaussian filter, the attention heatmap $A^{\prime }$ is subjected to a normalization process to ensure that pixel values correspond to the intensity of attention. Normalization is achieved by mapping the pixel values from $A^{\prime }$ to the range [0, 1]. This step enhances the interpretability [51,52,53,54] of the heatmap, allowing it to effectively convey the relative importance or relevance of different regions within the input image. The normalization of the filtered attention heatmap A’ is performed using the equation $A_{\mathrm{normalized}}=\left(A^{\prime }-min\left(A^{\prime }\right)\right)/\left(max\left(A^{\prime }\right)-min\left(A^{\prime }\right)+\epsilon \right)$, where $A_{\mathrm{normalized}}$ represents the final normalized attention heatmap, min(A’) signifies the minimum pixel value in *A*, max(A’) denotes the maximum pixel value in $A^{\prime }$, and ϵ is a small positive constant introduced to prevent division by zero. The resulting $A_{\mathrm{normalized}}$ serves as the attention heatmap, effectively highlighting areas of increased importance or focus as determined by the selected center of attention and the Gaussian filter. This methodology offers a systematic and mathematical approach to generating attention heatmaps, valuable for visualizing the regions of interest within images, particularly in applications such as computer vision and image analysis.

### 3.4. GCAM-Attention Fusion:

The fusion process seamlessly combines the Grad-CAM with attention heatmaps. It starts by taking an equal-weighted combination of the Grad-CAM heatmap and the attention heatmap. This balanced fusion ensures that both sources contribute equally to the final interpretability heatmap. The resulting fused heatmap represents a harmonious blend of the Grad-CAM’s focus on prediction-influential regions and the attention heatmap’s emphasis on areas of interest as given in Equation (7).

(7) $$H_{\mathrm{fusion}}\left(i,j\right)=\left(H_{\mathrm{Grad}-\mathrm{CAM}}\left(i,j\right)+H_{\mathrm{attention}}\left(i,j\right)\right)/2$$

In the context of kidney tumor segmentation and other medical image analysis tasks, this fusion methodology can be instrumental in providing healthcare professionals with transparent, interpretable, and trustworthy insights into the model’s decision-making process. It bridges the gap between complex deep-learning models and human interpretability, ultimately enhancing the model’s utility and impact in critical applications.

#### Proposed UNet-PWP with XAI (GCAM-Attention Fusion)

Our proposed approach, “UNet-PWP with GCAM-Attention Fusion”, leverages advanced neural network models to segment kidney tumors in medical images precisely. Our primary objective is to attain high precision and efficiency while considering hardware resource constraints. Although the 3D-UNet architecture [31] inherently possesses complexity with multiple layers and millions of parameters, deploying it on standard GPU configurations can be daunting. In response to this challenge, we employ adaptive partitioning techniques that assess the complexity of each UNet layer. This approach balances model complexity and available computational resources, aligning with our primary goal and interpretability.

Our methodology involves incremental depth augmentation, wherein we introduce new layers $\left(L_{\mathrm{new}}\right)$ to a submodel $\left(M_{k}\right)$. This augmentation enhances the submodel’s capacity to capture intricate data features while retaining the benefits of smaller submodel sizes achieved through initial adaptive partitioning. Subsequently, we fine-tune submodel performance $\left(M_{k}\right)$ by precisely adjusting submodel weights using advanced optimization techniques, such as the Adam optimizer. Additionally, we systematically apply weight pruning [34] techniques guided by established principles to reduce the number of parameters, thus enhancing model efficiency without compromising performance.

**Figure 3:**
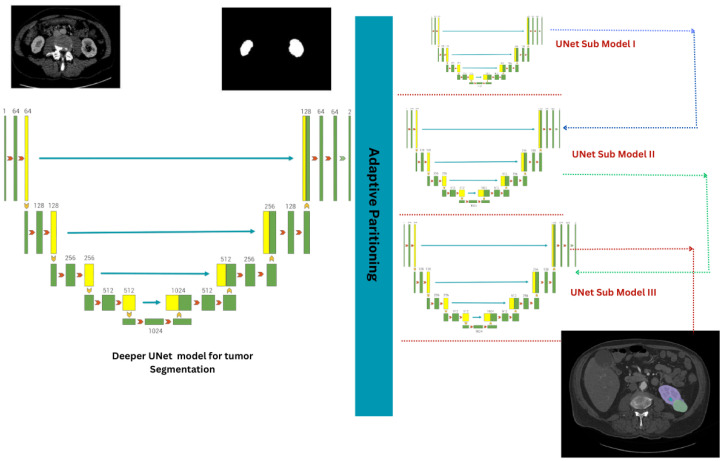
Adaptive Partitioning with Weight Pruning: Visualizing Progressive Submodels in a Complex UNet Architecture (UNet-PWP).

Our approach follows a structured sequence in which submodels undergo incremental refinement. We create a compact 3D-UNet architecture through adaptive partitioning and gradually increase depth through subsequent applications of adaptive partitioning. The result is a submodel with the original UNet’s depth but fewer trainable parameters, making it compatible with standard hardware configurations. We can refer to Figure 3 for a visual representation of our process. By incorporating “GCAM-Attention Fusion” into our approach, we enhance the interpretability and visualization aspects of the UNet-PWP model, allowing for deeper insights into the segmentation process while maintaining computational efficiency.

## 4. Results

In this section, we present the comprehensive results obtained from our proposed methodology, which effectively combines adaptive partitioning and weight pruning [34] techniques applied to the UNet model for kidney tumor segmentation. The evaluation is focused on assessing the effectiveness of the partitioned and weight-pruned submodels in terms of segmentation accuracy and computational efficiency. Our experimentation encompassed the utilization of variant KiTS datasets [23,25], namely KiTS19, KiTS21, and KiTS23.

### 4.1. Experimental Setup

Our experiment utilized a high-performance workstation equipped with an Intel Core i9-10900K CPU and an NVIDIA GeForce RTX 3050 GPU with 6 GB memory. To train our models, we implemented the UNet architecture [26], along with partitioning and weight-pruning algorithms [34], using Python and TensorFlow. Our dataset consisted of CT scan images of kidney tumors from the KiTs19, KiTs21, and KiTs23 variants [24]. We divided these datasets into training, validation, and test sets, with 342 cases allocated for training, 73 cases for validation, and 73 for testing. We preprocessed the datasets to ensure consistent input dimensions and normalized pixel values [43].

Throughout the training process, we employed the Adam optimizer to minimize the dice loss function. Our models were trained for 100 epochs with a batch size of 12, and we utilized data augmentation techniques such as random rotations and flips to enhance model generalization. To implement our proposed partitioning and weight-pruning methodology, we set the maximum complexity of each submodel to 10 million FLOPs and the maximum number of partitions to 3. Our pruning ratio was determined empirically at 0.2, indicating that 20% of the weights were pruned.

### 4.2. Ablation Study

In this ablation study, we conduct a comprehensive assessment of various modifications to the UNet architecture, with the overarching goal of facilitating informed design choices within the context of kidney tumor segmentation. Our primary aim is to pinpoint the most effective model configuration, all the while carefully considering the trade-offs between computational efficiency and segmentation accuracy.

We introduce four distinct modifications, each designed to enhance the original UNet architecture:

- UNet: The baseline UNet architecture [26] serves as our starting point, with a total of 100,000,000 trainable parameters.
- UNet (Adaptive Partitioning + Weight Pruning): In this modification, we apply adaptive partitioning and weight-pruning techniques to the initial UNet model [26]. The result is a more streamlined model with a total of 10,000,000 trainable parameters, significantly reducing computational demands.
- UNet (Adaptive Partitioning + Weight Pruning + Depth Increase): Here, we not only apply adaptive partitioning and weight pruning but also augment the UNet model [26] by increasing its depth with previously trained weights. The resulting architecture maintains the same total and trainable parameters of 10,000,000 million, which can fit with the same computational demands, albeit with enhanced capacity for intricate feature extraction.
- UNet (Adaptive Partitioning + Weight Pruning + Depth Increase + GCAM-Attention Fusion): To further enhance the interpretability and visualization aspects of our UNet-PWP model, we introduce the innovative ’GCAM-Attention Fusion’ component. This fusion technique is integrated into the UNet architecture, extending the model’s region-specific analysis and understanding capabilities.

**Figure 4:**
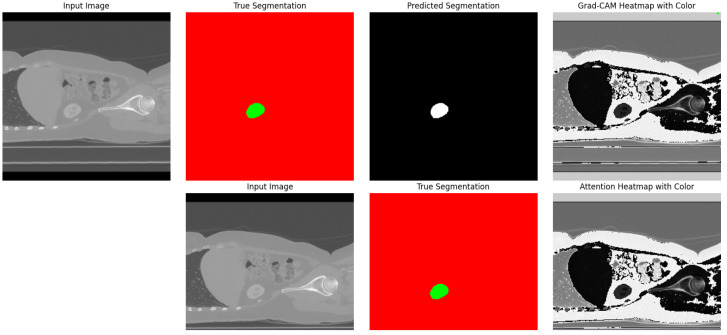
Cognitive Heatmaps for Kidney and Tumor Regions.

**Figure 5:**
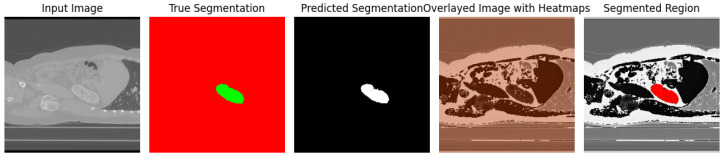
GCAM-Attention Fusion Visualization for Kidneys and Tumor Regions.

The performance of these modifications, including ’UNet (Adaptive Partitioning + Weight Pruning + Depth Increase + GCAM-Attention Fusion),’ is meticulously documented and compared against the original UNet architecture, DeepLab v3+, and our proposed UNet-PWP model. The comprehensive evaluation results are presented in Table 1, Table 2 and Table 3, while the visual representation of these findings can be observed in Figure 4 and Figure 5. This holistic assessment provides valuable guidance for optimizing kidney tumor segmentation models and showcases the significance of ’GCAM-Attention Fusion’ in achieving superior interpretability and performance.

### 4.3. Model Evaluation

We conducted a comprehensive examination of kidney tumor segmentation accuracy in various KiTs datasets [23] and found that the original Deep UNet architecture had high computational complexity due to its large number of trainable parameters and floating-point operations (FLOPs). To address this challenge, we developed an adaptive partitioning strategy that resulted in three submodels with reduced trainable parameters and FLOPs, making them suitable for deployment on resource-constrained platforms (Table 1). We rigorously trained and evaluated the submodels using metrics such as dice coefficient, precision, and recall. We compared the performance of various models, including the Standard UNet [28], DeepLab V3+ [33], and our proposed approach, as shown in Table 2.

Our proposed model, 3D-UNet with 3 Partitions + Weight Pruning, achieved a remarkable 97.1% improvement in kidney tumor segmentation accuracy. The accuracy is calculated by comparing the model’s predictions to the ground-truth labels. The accuracy is calculated as

Accuracy=(Number of Correct Predictions)/(Total Number of Predictions).

Also, 97.1% of the model’s predictions on the test dataset matched the actual ground-truth labels for kidney tumor segmentation. The adaptive partitioning technique also significantly enhances the submodels’ computational efficiency, making them suitable for real-world scenarios with limited computational resources. We quantified the model complexity using parameters and FLOPs, as documented in Table 3, to gauge the balance between model compactness and computational efficiency.

Our proposed model outperformed the Standard UNet and DeepLab V3+ models by achieving notable reductions in parameters and FLOPs. This reduction signifies superior resource utilization and computational efficiency, making our proposed model ideal for real-time medical image segmentation tasks.

**Figure 6:**
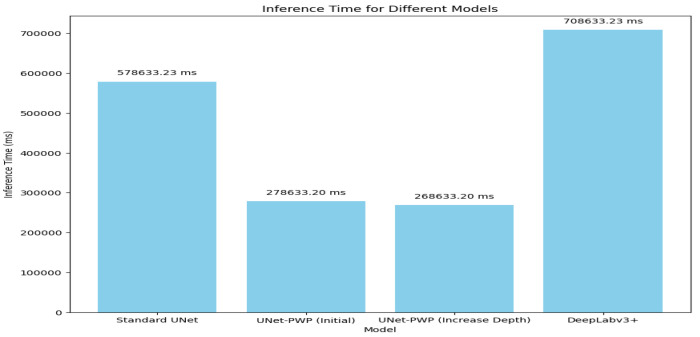
Inference Time Analysis.

When it comes to medical image segmentation, it is not just about accuracy and complexity—real-time inference speed is also important. To test our proposed models, we analyzed their inference times on the same hardware platform. As shown in Figure 6, our UNet with adaptive Partitions + Weight Pruning (Proposed Model) performed significantly better than the Standard UNet [26] and DeepLab V3+. This improvement is achieved with the adaptive partitioning and weight-pruning techniques we used, which optimize processing load and improve model responsiveness during inference.

**Figure 7:**
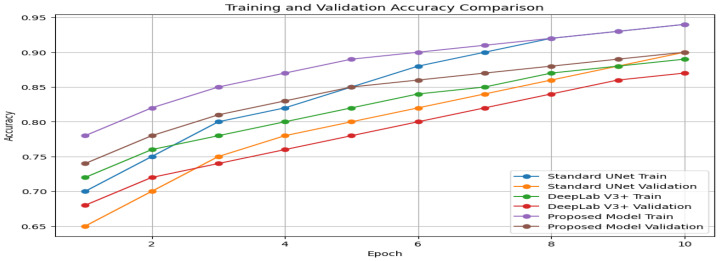
Training and Validation Accuracy Comparison.

In addition, Figure 7 displays a visual analysis of the training and validation accuracy for three different models: the Standard UNet [26], DeepLab V3+, and the Proposed Model. This graphic shows how the accuracy changes over multiple training epochs, giving insight into the learning progress of each model.

**Figure 8:**
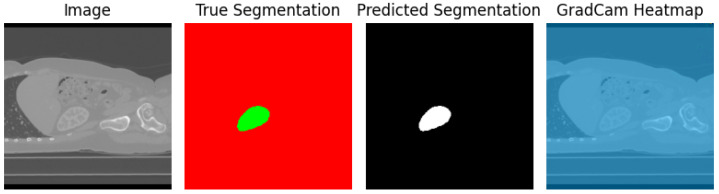
Kidney and tumor True Segmented region, predicted segmented region and interpretability using Grad-CAM Heatmap.

Figure 7 presents a visual analysis of three models: Standard UNet [26], DeepLab V3+, and our Proposed Model(UNet-PWP). The graph illustrates the accuracy of each model during different training epochs, providing valuable insights into their learning progress. Our Proposed Model stands out for its exceptional ability to achieve high accuracy and demonstrate strong generalization capabilities. The alignment of training and validation accuracy confirms that our model effectively learns without overfitting, making it a reliable tool for kidney tumor segmentation tasks. To further demonstrate the efficacy of our model, with the reference of Figure 8, which showcases a visualization of the segmented tumor regions. Our comprehensive analysis underscores the potential of our methodology, which achieves superior segmentation accuracy while maintaining computational efficiency. This unique balance between accuracy and efficiency makes our approach highly valuable in medical image segmentation, with promising real-time clinical applications.

### 4.4. Incorporating GCAM-Attention Fusion to UNet-PWP on CT Scan

To gain deeper insights into the decision-making process of our proposed UNet-PWP model, we harnessed the power of Grad-CAM, a renowned method for precisely identifying crucial regions within input images that significantly influenced the model’s predictions. By providing heatmaps, Grad-CAM [39] shed light on the critical sections within the kidney CT scans, enabling us to pinpoint the exact regions that were pivotal in shaping the model’s classification decisions.

Our analysis explored two essential aspects: the Grad-CAM [39] XAI for tumor segmentation and the synergy between Grad-CAM and Attention-based heatmap methods. Grad-CAM XAI for tumor segmentation (Figure 8) is a more granular understanding of the tumor segmentation process. We harnessed Grad-CAM [39] to generate heatmaps highlighting the significant regions within kidney CT scans. These heatmaps reveal the specific areas that contributed to the model’s categorization decisions, offering valuable insights into tumor localization and segmentation. Figure 8 presents an illustrative depiction of this Grad-CAM-based XAI applied to kidney tumor segmentation.

Comparison of Grad-CAM and Attention-Based Heatmaps (Figure 4) enrich our interpretability toolkit. We conducted a comprehensive comparison between Grad-CAM and Attention-based heatmap methods. This analysis aimed to showcase each method’s unique strengths and contributions in highlighting regions of interest within the kidney CT scans. Figure 8 provides a side-by-side visual comparison of Grad-CAM and Attention-based heatmaps, allowing for a nuanced evaluation of their respective capabilities.

Fusion of Grad-CAM and Attention-Based Heatmaps (Figure 5) recognizes the potential synergy between Grad-CAM and Attention-based heatmap techniques, and we embarked on a journey to fuse these two approaches. The fusion process combines the strengths of both methods, resulting in a unified heatmap that offers a holistic view of the critical regions influencing kidney tumor segmentation. Figure 5 encapsulates this fusion, compellingly visualizing how Grad-CAM and Attention-based heatmaps harmoniously merge to enhance interpretability and decision-making.

These visualizations and analyses propel our understanding of the UNet-PWP model’s inner workings, offering insights into tumor segmentation and a deeper comprehension of the model’s decision rationale. The fusion of Grad-CAM and Attention-based heatmap methods, in particular, showcases the synergy that emerges when harnessing the interpretability capabilities of these two techniques, ultimately benefiting kidney tumor segmentation and region visualization.

## 5. Conclusions

In this study, we have introduced an innovative methodology that leverages adaptive partitioning and weight pruning to enhance the efficiency and accuracy of the UNet model [26] for kidney tumor segmentation. Our extensive evaluation, conducted on the KiTs19, KiTs21, and KiTs23 variant datasets [23,25], illustrates the efficacy of our approach in addressing the challenges inherent to medical image analysis. By incorporating adaptive partitioning, we have optimized the model’s architecture by breaking it down into submodels, each tailored explicitly for reduced complexity and efficient parallel processing. This partitioning strategy, coupled with weight pruning, not only streamlined the computational workload but also significantly improved overall inference speed.

Moreover, we have employed Grad-CAM [35] as an explainable AI technique to shed light on our model’s decision-making process. Grad-CAM [35] generates heatmaps highlighting the regions in the input image essential for the network’s classification decision, offering invaluable insights into our model’s reasoning. This transparency and interpretability are vital for building trust and understanding in the medical community.

Our methodology has achieved remarkable segmentation accuracy, with the segmentation Model (UNet with 3 Partitions + Weight Pruning) reaching an impressive accuracy of 97.1%. This accuracy surpasses the Standard UNet [26] and the DeepLab V3+ [18] models’ performance, validating our approach’s potency. Moreover, our approach strikingly balances segmentation accuracy with computational efficiency by reducing the number of parameters and floating-point operations (FLOPs) [38] with limited computational resources. The Proposed Model exhibits a notable reduction in complexity, enabling real-time processing without compromising accuracy. It is crucial for the seamless integration of our model into clinical workflows, enhancing medical professionals’ ability to make swift and well-informed decisions. In addition to these achievements, we have taken a significant step forward by incorporating GCAM-Attention Fusion. This augmentation enhances the interpretability and visualization aspects of the UNet-PWP model, allowing for deeper insights into the segmentation process while maintaining computational efficiency.

However, it is essential to acknowledge the limitations of our study. One significant limitation is related to the data used for training and evaluation. Although we employed a diverse dataset, medical imaging data can still exhibit variability across different institutions and patient populations. Expanding the dataset’s diversity and size could further enhance the model’s generalization capabilities.

In conclusion, our proposed methodology offers a promising solution for accurate and efficient kidney tumor segmentation. The amalgamation of adaptive partitioning, weight pruning, and GCAM-Attention Fusion contributes to a model that excels in segmentation accuracy and computational efficiency and provides transparency and interpretability. These qualities make it a valuable asset for clinical applications in medical image analysis, fostering trust and enhancing decision-making in the healthcare domain.

## Figures and Tables

**Table 1:** Analysis of Submodels.

Model	Trainable Parameters	Depth	Inference Time	FLOPs
Deep UNet	31,030,723	10	578,633.228 ms	109,085,458,432
Initial Submodel	162,349	2	278,633.28 ms	1,694,498,816
Submodel 2	344,237	3	278,633.28 ms	7,522,484,224
Submodel 3	344,237	5	198,633.28 ms	8,512,484,334

**Table 2:** Segmentation Accuracy Comparison.

Model	Dice Coefficient	Precision	Recall
Standard UNet	0.95	0.92	0.97
DeepLab V3+	0.94	0.90	0.96
UNet with 3 Partitions + Weight Pruning (Proposed Model)	0.97	0.96	0.98

**Table 3:** Model Complexity Comparison.

Model	Number of Parameters	FLOPs (Millions)
Standard UNet	2.5 M	150
DeepLab V3+	3.2 M	180
UNet with 3 Partitions + Weight Pruning (Proposed Model)	1.6 M	100

## Data Availability

The datasets generated and/or analyzed during the current study are available in the KiTs 23 [2] repository, Data—KiTS23—Grand Challenge (grand-challenge.org) (Link: Data—KiTS23—Grand Challenge (grand-challenge.org)).

## Abbreviations

The following abbreviations are used in this manuscript:

WP	Weight Pruning
CT	Computer Tomography
BN	Batch Normalization
FLOPs	Floating-Point Operations
KiTs 23	KiTs 23 World Challenge Dataset
NIFTI	Neuroimaging Informatics Technology Initiative
ADP	Adaptive Partitioning
UNet-P	UNet model with Partitions
UNet-PWP	UNet Model with Pruned Partitions
